# Chronic Obstructive Pulmonary Disease and Altered Risk of Lung Cancer in a Population-Based Case-Control Study

**DOI:** 10.1371/journal.pone.0007380

**Published:** 2009-10-08

**Authors:** Jill Koshiol, Melissa Rotunno, Dario Consonni, Angela Cecilia Pesatori, Sara De Matteis, Alisa M. Goldstein, Anil K. Chaturvedi, Sholom Wacholder, Maria Teresa Landi, Jay H. Lubin, Neil E. Caporaso

**Affiliations:** 1 Division of Cancer Epidemiology and Genetics, National Cancer Institute, National Institutes of Health, Department of Health and Human Services, Bethesda, Maryland, United States of America; 2 EPOCA Research Center, Department of Occupational and Environmental Health, Università degli Studi di Milano, and Epidemiology Unit, Fondazione IRCCS Ospedale Maggiore Policlinico, Mangiagalli e Regina Elena, Milan, Italy; Johns Hopkins School of Medicine, United States of America

## Abstract

**Background:**

Chronic obstructive pulmonary disease (COPD) has been consistently associated with increased risk of lung cancer. However, previous studies have had limited ability to determine whether the association is due to smoking.

**Methodology/Principal Findings:**

The Environment And Genetics in Lung cancer Etiology (EAGLE) population-based case-control study recruited 2100 cases and 2120 controls, of whom 1934 cases and 2108 controls reported about diagnosis of chronic bronchitis, emphysema, COPD (chronic bronchitis and/or emphysema), or asthma more than 1 year before enrollment. We estimated odds ratios (OR) and 95% confidence intervals (CI) using logistic regression. After adjustment for smoking, other previous lung diseases, and study design variables, lung cancer risk was elevated among individuals with a history of chronic bronchitis (OR = 2.0, 95% CI = 1.5–2.5), emphysema (OR = 1.9, 95% CI = 1.4–2.8), or COPD (OR = 2.5, 95% CI = 2.0–3.1). Among current smokers, association between chronic bronchitis and lung cancer was strongest among lighter smokers. Asthma was associated with a decreased risk of lung cancer in males (OR = 0.48, 95% CI = 0.30–0.78).

**Conclusions/Significance:**

These results suggest that the associations of personal history of chronic bronchitis, emphysema, and COPD with increased risk of lung cancer are not entirely due to smoking. Inflammatory processes may both contribute to COPD and be important for lung carcinogenesis.

## Introduction

Every year, over 1 million people die from lung cancer worldwide [1]. Although cigarette smoking is the primary etiologic agent in 85–90% of all lung cancers [2], only 10–15% of active smokers develop lung cancer [3]. In addition, lung cancer is the seventh most common cause of cancer death worldwide in never smokers [4]. While risk factors such as family history of lung cancer, occupational carcinogens, and radon account for some increased risk, the etiology of lung cancer in this population remains poorly understood [4]. Associations between nicotinic receptors and lung cancer from genome-wide association studies [5]–[7] only account for a small component of the increased risk, suggesting that additional factors must be involved in lung carcinogenesis.

Chronic obstructive pulmonary disease (COPD) has been suggested as a risk factor for lung cancer. COPD can be exacerbated by pulmonary infections [8], [9] that cause inflammation, which contributes to lung carcinogenesis [10], and carcinogenesis in general [11], by generating reactive oxygen or nitrogen species, increasing cellular proliferation, upregulating antiapoptotic pathways, and stimulating angiogenesis [10]. Infections may also promote airway remodeling that could enhance carcinogenesis [12]. Although COPD is strongly and consistently associated with lung cancer [13]–[16], the degree to which the association between COPD and lung cancer is due to smoking or other factors remains unclear [3]. Few studies have had appropriate data and sufficient cases to evaluate COPD and lung cancer by histology and time since diagnosis of previous COPD.

The Environment And Genetics in Lung cancer Etiology population-based case-control study (EAGLE) was specifically designed to evaluate comprehensively a variety of risk factors for lung cancer. With over 2000 cases, EAGLE allows evaluation of the association of COPD and lung cancer by smoking status, histology, gender, and time since COPD diagnosis.

## Materials and Methods

The EAGLE study has previously been described [17]. In brief, EAGLE is a large population-based study of 2100 consecutive incident lung cancer cases and 2120 controls from the Lombardy region of northern Italy. Cases were enrolled from 13 hospitals in 216 municipalities including 5 large cities (Milan, Monza, Brescia, Pavia, and Varese). Healthy controls were randomly sampled from the Regional Health Service database and frequency matched to cases by age, sex, and area of residence. The participation rates (number of subjects who agreed to participate/eligible subjects) were 86.6% for cases and 72.4% for controls. Each participant provided written informed consent. The study was approved by the Institutional Review Board (IRB) of each participating hospital and university in Italy and by the National Cancer Institute, Bethesda, MD.

We defined COPD as reporting a diagnosis of chronic bronchitis and/or emphysema. We chose not to include asthma in the definition of COPD since asthma is not strongly associated with smoking and often develops in childhood [18], [19], whereas chronic bronchitis and emphysema are strongly associated with smoking, occur more commonly with increasing age, and often occur together [20]–[22]. We included asthma separately since historically it has been considered a component of COPD [23].

Participants were asked (in Italian) through a computer-assisted personal interview (CAPI) “whether a doctor ever told you more than one year ago that you had any of these conditions: chronic bronchitis, emphysema, asthma” and “How old were you when this condition was first diagnosed?” We evaluated chronic bronchitis and emphysema both independently and jointly. We calculated latency for each condition as the difference between study age (age at first diagnosis of lung cancer or interview) and age at first diagnosis of previous lung disease. Ten cases and four controls who provided a date of previous chronic bronchitis, emphysema, or asthma less than one year before study entry were excluded, leaving 2091 cases and 2116 controls. Of these, 1934 (92.5%) cases and 2108 (99.6%) controls provided data on chronic bronchitis, emphysema, or asthma. These percentages are similar to the overall CAPI completion rates for cases (92.6%) and controls (99.8%).

Lung cancer was diagnosed according to standard clinical criteria with pathologic confirmation from surgery, biopsy, or cytology samples in approximately 95% of cases. The remaining cases were confirmed through clinical history and imaging [17]. Main analyses included all primary lung cancer cases regardless of histological type. Histology-specific analyses were restricted to adenocarcinoma, squamous cell carcinoma, large cell carcinoma, or small cell carcinoma. Tumor histology was defined using the WHO Histological Typing of Lung and Pleural Tumors (1999).

We used unconditional binary logistic regression to calculate odds ratios (ORs) and 95% confidence intervals (CIs) for the association of chronic bronchitis, emphysema, COPD, and asthma with lung cancer and polytomous logistic regression to calculate ORs and 95% CIs by histological type. All models included the design variables (study age, gender, and region). Potential confounders, including smoking (e.g., smoking intensity (average packs per day), time since last smoking quit attempt and entry into EAGLE), demographic/socioeconomic variables (e.g., education, marital status), and other factors, were evaluated through backwards modeling using chronic bronchitis as the main exposure. Since the removal of continuous pack-years, emphysema, and pneumonia changed the beta coefficient for chronic bronchitis by more than 10%, they were retained in the adjusted models. In accord with several recent studies [24]–[26], we selected pack-years and smoking intensity to adjust for smoking exposure; however, models using other closely related smoking metrics (smoking duration; time since quitting smoking; age at initiation of cigarette smoking; environmental tobacco smoke in childhood, adulthood at work, or adulthood at home; and other tobacco smoking) produced almost identical results. Chronic bronchitis changed the beta coefficient for emphysema by more than 10% and thus was included in multivariate models where chronic bronchitis was not the main effect or a component of the main effect (i.e., COPD).

Smoking, gender, and latency were evaluated as effect-measure modifiers using likelihood ratio tests (LRTs) for interaction on the multiplicative scale adjusted as described above. Differences in ORs for separate histological types were evaluated with the Wald test for homogeneity.

## Results

Compared to controls, cases tended to be less educated, less likely to be married or cohabitating, and more likely to be current smokers [Table 1]. Among smokers, the mean smoking intensity (average packs per day) was 1.1 (standard deviation (SD)  = 0.5) among cases and 0.8 (SD = 0.5) among controls, and the mean pack-years was 48.6 (SD = 27.9) among cases and 27.3 (SD = 22.1) among controls. Among cases, the mean age at diagnosis of chronic bronchitis was 45.0 years (median  = 48, range  = 0–78) in cases and 56.4 years (median  = 61, range  = 3–77) in controls (t-test for difference in group means p-value <0.001), of emphysema was 54.2 years (median 57, range  = 6–77) in cases and 56.3 years (median 58, range  = 18–78) in controls (t-test p-value  = 0.3), of COPD was 46.4 years (median  = 50, range  = 0–78) in cases and 56.4 years (median  = 60, range  = 3–78) in controls (t-test p-value <0.001), and of asthma was 39.1 years (median  = 44, range  = 0–75) in cases and 41.6 years (median  = 49, range  = 1–77) in controls (t-test p-value  = 0.5).

**Table 1:** Distribution of cases and controls in the Environment And Genetics in Lung cancer Etiology study who provided data on chronic bronchitis, emphysema, or asthma diagnosed at least one year prior to study entry.

		Cases	Controls
Characteristic	Sub-category	N	%	N	%
Study age					
	<60	420	21.7	543	25.8
	60–<65	339	17.5	370	17.6
	65–<70	429	22.2	486	23.1
	> = 70	746	38.6	709	33.6
Gender					
	Male	1528	79.0	1610	76.4
	Female	406	21.0	498	23.6
Education*					
	None	112	5.8	90	4.3
	Elementary	748	38.7	570	27.0
	Middle school	554	28.7	612	29.0
	High school	418	21.6	574	27.2
	University	101	5.2	262	12.4
Marital status					
	Married/cohabitating	1491	77.1	1741	82.6
	Not married/cohabitating	443	22.9	367	17.4
Smoking status*					
	Never	133	6.9	678	32.2
	Former	833	43.1	905	43.0
	Current	968	50.1	524	24.9
Chronic bronchitis*					
	No	1466	77.7	1943	93.1
	Yes	421	22.3	144	6.9
Emphysema*					
	No	1699	89.3	2038	97.0
	Yes	203	10.7	62	3.0
COPD* †					
	No	1372	72.9	1908	91.6
	Yes	509	27.1	174	8.4
Asthma*					
	No	1836	95.9	2005	95.3
	Yes	78	4.1	99	4.7

* Does not sum to total due to missing values.

† COPD = chronic obstructive pulmonary disease (chronic bronchitis and/or emphysema).

After adjustment for smoking and other factors, chronic bronchitis, emphysema, and COPD were associated with an approximately two-fold increased risk of lung cancer [Table 2]. Results were similar when restricted to individuals diagnosed with chronic bronchitis, emphysema, or COPD at or above age 18 (data not shown). Evaluating chronic bronchitis and emphysema as a combined variable, the OR for having both chronic bronchitis and emphysema (2.5, 95% CI = 1.6–4.0) was similar to the ORs for having chronic bronchitis only (2.3, 95% CI = 1.8–3.0) or emphysema only (2.9, 95% CI = 1.8–4.6). Given that the OR for having both chronic bronchitis and emphysema was no stronger than having chronic bronchitis or emphysema separately, chronic bronchitis and emphysema were maintained as separate variables. In a subset of cases with spirometry data available, self report-based COPD was strongly associated spirometry-based COPD [27] (OR = 3.0, 95% CI = 2.1–4.3).

**Table 2:** Associations of lung cancer with lung disease diagnosed at least one year prior in the Environment And Genetics in Lung cancer Etiology case-control study.

Lung disease	Sub-category	Minimally Adjusted OR (95% CI)*	Fully Adjusted OR (95% CI)†
Chronic bronchitis			
	No	1.0	1.0
	Yes	3.8 (3.1–4.7)	2.0 (1.6–2.5)
Emphysema			
	No	1.0	1.0
	Yes	3.8 (2.8–5.1)	1.9 (1.4–2.7)
COPD‡			
	No	1.0	1.0
	Yes	4.1 (3.4–4.9)	2.5 (2.0–3.1)
Asthma in males			
	No	1.0	1.0
	Yes	0.77 (0.54–1.1)	0.48 (0.30–0.78)
Asthma in females			
	No	1.0	1.0
	Yes	01.1 (0.64–2.0)	1.1 (0.57–2.3)

* OR = odds ratio, CI = confidence interval. Adjusted for study age, sex, and region.

† OR = odds ratio, CI = confidence interval. Adjusted for study age, sex, region, pack-years, amount of cigarette smoking (average packs/day), bronchitis (unless main effect or COPD), emphysema (unless main effect or COPD), and pneumonia.

‡ COPD = chronic obstructive pulmonary disease (chronic bronchitis and/or emphysema).

Although gender did not modify the association of lung cancer with chronic bronchitis, emphysema, or COPD (LRT p-values  = 0.4, 0.3, 0.6, respectively), the OR for asthma among males was 0.48 (95% CI = 0.30–0.78) and among females was 1.1 (95% CI = 0.57–2.3) (LRT p-value for interaction  = 0.03). Among men, even the minimally adjusted OR suggested an inverse association, which was substantially strengthened after accounting for chronic bronchitis and emphysema, both of which increased the risk of lung cancer [Table 2]. Among women there was no association regardless of adjustment. Since only 48 women (23 cases) reported asthma diagnosis, additional analyses focused on men with more limited analyses in women.

The ORs for chronic bronchitis and asthma did not vary by smoking status [Table 3]. We observed an increased risk with emphysema only among smokers, although only two never-smoking cases had emphysema, limiting power to detect differences by smoking status (LRT p-value = 0.3). Similarly, the strongest effect of COPD was seen in smokers, although the LRT p-value was 0.4. Restricted to current smokers, the strength of the association between chronic bronchitis and lung cancer decreased with increasing pack-years, smoking intensity, and smoking duration [Table 4] (LRT p-values for continuous pack-years and smoking intensity <0.001, smoking duration 0.02). The trends for COPD were similar to those for chronic bronchitis. Emphysema and asthma in males showed no clear pattern by pack-years, smoking intensity, and smoking duration. There were too few women with asthma for extended analyses by smoking status.

**Table 3:** Associations of lung cancer with lung disease diagnosed at least one year prior and lung cancer in the Environment And Genetics in Lung cancer Etiology case-control study, stratified by smoking status.

Lung Disease	Sub-category	Never	Former	Current	LRT‡ *p*-value
Chronic bronchitis					
	Cases, N yes/total (%)	7/131 (5.3)	185/813 (22.8)	229/943 (24.3)	0.6
	Controls, N yes/total (%)	20/673 (3.0)	70/895 (7.8)	54/519 (10.4)	
	OR (95% CI)*	2.0 (0.75–5.5)	2.1 (1.5–2.9)	1.8 (1.2–2.5)	
Emphysema					
	Cases, N yes/total (%)	2/131 (1.5)	96/819 (11.7)	105/952 (11.0)	0.3
	Controls, N yes/total (%)	13/677 (1.9)	32/900 (3.6)	17/522 (3.3)	
	OR (95% CI)*	0.69 (0.14–3.5)	2.0 (1.3–3.3)	2.2 (1.2–3.9)	
COPD†					
	Cases, N yes/total (%)	8/130 (6.2)	224/808 (27.7)	277/943 (29.4)	0.4
	Controls, N yes/total (%)	29/672 (4.3)	84/893 (9.4)	61/517 (11.8)	
	OR (95% CI)*	1.5 (0.60–3.6)	2.7 (2.0–3.7)	2.3 (1.7–3.2)	
Asthma in males∥					
	Cases, N yes/total (%)	0/29 (0.0)	28/714 (3.9)	27/770 (3.5)	0.3
	Controls, N yes/total (%)	22/395 (5.6)	39/794 (4.9)	12/418 (2.9)	
	OR (95% CI)*	0	0.44 (0.24–0.82)	0.70 (0.31–1.6)	

* OR = odds ratio, CI = confidence interval. Adjusted for study age, sex, region, bronchitis (unless main effect or COPD), emphysema (unless main effect or COPD) and pneumonia for never smokers and also pack-years and amount of cigarette smoking (average packs/day) for smokers.

† COPD = chronic obstructive pulmonary disease (chronic bronchitis and/or emphysema).

‡ LRT = likelihood ratio test.

∥ OR = 0.76 (95% CI = 0.26–2.2) in female never smokers and 1.7 (95% CI = 0.59–5.1) in female ever smokers, LRT p-value  = 0.2.

**Table 4:** Associations of lung cancer with lung disease diagnosed at least one year prior and lung cancer in the Environment And Genetics in Lung cancer Etiology case-control study restricted to current smokers (968 cases, 524 controls) and stratified by smoking status (tertiles among controls).

		Chronic bronchitis	Emphysema	COPD‡	Asthma in males
Smoking Variable	Tertile	OR (95% CI)*	LRT† *p*-value	OR (95% CI)*	LRT† *p*-value	OR (95% CI)*	LRT† *p*-value	OR (95% CI)*	LRT† *p*-value
Pack-years									
	<14.4	5.0 (1.5–16.4)	<0.001	3.5 (0.26–45.4)	0.05	5.0 (1.7–15.2)	<0.001	0.26 (0.02–4.2)	0.2
	14.4–<34.5	2.1 (1.1–4.1)		1.5 (0.52–4.0)		2.4 (1.3–4.3)		1.5 (0.14–17.2)	
	> = 34.5	1.3 (0.81–2.0)		2.7 (1.3–5.6)		1.9 (1.2–3.0)		0.70 (0.25–1.9)	
Intensity									
(average	<0.5	7.1 (2.0–25.5)	<0.001	2.5 (0.44–14.7)	0.07	5.6 (1.9–16.4)	<0.001	1.2 (0.10–13.2)	0.3
packs/day)	0.5–<1	1.3 (0.70–2.6)		3.2 (1.0–9.8)		2.1 (1.2–3.9)		0.49 (0.05–5.3)	
	> = 1	1.6 (0.99–2.5)		1.8 (0.88–3.7)		2.0 (1.3–3.1)		0.88 (0.32–2.4)	
Duration									
(years)	<26	4.5 (1.5–13.2)	0.02	2.3 (0.20–28.0)	0.8	6.2 (2.1–17.8)	0.08	0.36 (0.05–2.8)	0.5
	26–<40	3.3 (1.6–6.8)		1.2 (0.39–4.0)		3.1 (1.6–6.1)		1.1 (0.16–7.5)	
	> = 40	1.1 (0.70–1.7)		2.7 (1.4–5.5)		1.7 (1.1–2.6)		0.77 (0.26–2.3)	

* OR = odds ratio, CI = confidence interval. Adjusted for study age, sex, region, bronchitis (unless main effect or COPD), emphysema (unless main effect or COPD), pneumonia, pack-years, and amount of cigarette smoking (average packs/day).

† LRT = likelihood ratio test, using continuous smoking variables.

‡ COPD = chronic obstructive pulmonary disease (chronic bronchitis and/or emphysema).

Risk of lung cancer increased with time since diagnosis of chronic bronchitis (LRT p-value  = 0.002) and COPD (LRT p-value  = 0.007) but did not consistently increase or decrease with time since diagnosis of emphysema (LRT p-value  = 0.2) or asthma among males (LRT p-value  = 0.9) [Table 5]. The asthma in males was consistently associated with a decreased risk of lung cancer for asthma diagnosed more than five years prior, however. Among women, the OR for asthma diagnosed 1–5 years prior to lung cancer or enrollment was 0.50 (95% CI = 0.09–2.9) and for asthma diagnosed >5 years prior was 1.7 (95% CI = 0.75–4.0, LRT p-value = 0.4). The distribution of ever/never smoking, time since last quit smoking, and family history of lung cancer varied little by latency (data not shown). Although latency and age at diagnosis are highly correlated, we also evaluated the association of age at diagnosis of chronic bronchitis or COPD with risk of lung cancer since the mean age at diagnosis of chronic bronchitis and COPD differed for cases and controls. The age at diagnosis results mirrored the latency results. Restricting to subjects with known age at diagnosis, later age at diagnosis (and thus likely shorter latency) was associated with decreased risk of lung cancer for both chronic bronchitis (OR = 0.95, 95% CI = 0.93–0.97) and COPD (OR = 0.95, 95% CI = 0.93–0.97).

**Table 5:** Distributions of cases and controls from the Environment And Genetics in Lung cancer Etiology study and associations of lung cancer with lung disease diagnosed at least one year prior, stratified by time since diagnosis of lung disease (latency).

Lung Disease	Latency (Years)	N Cases	N Controls	OR (95% CI)*
Chronic bronchitis†				
	None	1431	1912	1.0
	1–5	57	35	1.1 (0.70–1.9)
	>5–10	69	21	1.9 (1.1–3.4)
	>10–15	40	20	1.7 (0.91–3.2)
	>15	197	39	3.9 (2.6–5.8)
Emphysema†				
	None	1627	1993	1.0
	1–5	44	13	2.3 (1.2–4.7)
	>5–10	30	14	1.2 (0.55–2.4)
	>10–15	41	4	4.9 (1.7–14.5)
	>15	61	21	1.8 (1.0–3.2)
COPD† ‡				
	None	1348	1883	1.0
	1–5	77	43	1.5 (0.95–2.3)
	>5–10	73	25	2.6 (1.6–4.4)
	>10–15	54	21	2.4 (1.4–4.3)
	>15	222	50	4.1 (2.9–5.9)
Asthma in males† ^∥^				
	None	1376	1493	1.0
	1–5	10	5	1.0 (0.26–3.8)
	>5–10	7	8	0.49 (0.15–1.6)
	>10–15	5	8	0.40 (0.11–1.5)
	>15	22	40	0.44 (0.23–0.85)

* OR = odds ratio, CI = confidence interval. Adjusted for study age, gender, region, pack-years, amount of cigarette smoking, bronchitis (unless main effect or COPD), emphysema (unless main effect or COPD), and pneumonia.

† Does not sum to total due to missing values in multivariate analysis.

‡ COPD = chronic obstructive pulmonary disease (chronic bronchitis and/or emphysema).

∥ OR for females with asthma diagnosed 1–5 years prior  = 0.50 (95% CI = 0.09–2.9) and for asthma diagnosed >5 years prior  = 1.7 (95% CI = 0.75–4.0, LRT p-value  = 0.4).

The associations of lung cancer with chronic bronchitis, emphysema, COPD, asthma in men, and asthma in women did not vary by histology (Wald p-value  = 0.4, 0.4, 0.7, 0.6, 0.7, respectively), although numbers were small for some histology categories. We also stratified by high and low pack-years for adenocarcinoma separately because this histologic subgroup exhibits demographic, smoking-related [4], [28], and molecular [29], [30] differences compared with other histologies. Among ever smokers, the OR for chronic bronchitis was 1.8 (95% CI = 0.91–3.7) for adenocarcinoma and ≤24 pack-years, 1.7 (95% CI = 1.2–2.4) for adenocarcinoma and >24 pack-years, 3.7 (95% CI = 3.8–7.6) for other histologies (squamous cell, large cell, and small cell carcinoma) and ≤24 pack-years, 1.6 (95% CI = 1.1–2.2) for other histologies and >24 pack-years (LRT p-value  = 0.02). The associations of emphysema, COPD, and asthma and risk of adenocarcinoma and other histologies varied little by high or low pack-years (LRT p-values  = 0.4, 0.2, and 0.2, respectively).

## Discussion

In this large study of chronic obstructive pulmonary disease (COPD) and lung cancer, we found that history of chronic bronchitis, emphysema, and COPD were associated with increased risk of lung cancer. The risk in patients with both chronic bronchitis and emphysema was similar to that in patients with only chronic bronchitis or emphysema. Previous asthma was associated with decreased risk of lung cancer in males. Additional adjustment for smoking beyond pack-years and smoking intensity did not materially change these results.

While there is strong and compelling evidence for associations between COPD and lung cancer [14]–[16], [31], some have argued that this association may be largely due to smoking, even after adjustment [32]. However, several lines of evidence suggest that the association between COPD and lung cancer may not be entirely due to smoking. Family history of chronic bronchitis and emphysema are associated with increased risk of lung cancer [33]. In addition, COPD is associated with lung cancer in never-smokers [31]. A recent study estimated that COPD accounts for 10% of lung cancer cases among never smokers and 12% among heavy smokers [34]. We found that even restricted to adenocarcinoma, which is more common among non-smokers, particularly women [4], COPD remained strongly associated with lung cancer. In addition, the association between chronic bronchitis and lung cancer was stronger among smokers with lower pack-years, smoking intensity, and smoking duration. The stronger association among lighter smokers may suggest that chronic bronchitis and smoking share some molecular features, possibly involving inflammation. We can speculate that among lighter smokers both chronic bronchitis and smoking strongly contribute, while at heavier smoking levels some “saturation” occurs so that the contribution of chronic bronchitis to lung cancer appears less prominent. To fully evaluate these factors in concert with molecular and genetic markers (which for example, may assess inflammation and associated genes), larger studies in consortial settings will be required. Taken together, our data suggest that COPD contributes independently to lung cancer risk and are concordant with other evidence that some proportion of COPD cases develop lung cancer because of COPD itself, rather than because of its association with smoking. One potential mechanism is lung infections leading to inflammation, chronic immune stimulation [35], COPD exacerbation, and accelerated lung function decline [36]. Although our data suggest that COPD may contribute independently to the risk of lung cancer, the proportion of never-smoking cases in EAGLE was small, underscoring the need for additional analyses among never smokers.

Previous studies have not found consistent patterns in the association of chronic bronchitis and emphysema with lung cancer by latency [37]–[40]. Our finding that chronic bronchitis was most strongly associated with lung cancer among people who were diagnosed with chronic bronchitis more than 15 years prior to diagnosis of lung cancer is noteworthy because it rules out the possibility that the association with chronic bronchitis is due to reverse causality, i.e., the diagnosis of chronic bronchitis due to underlying lung cancer. Moreover, it suggests that chronic bronchitis may act in an early stage of lung carcinogenesis.

We found that asthma was associated with a decreased risk of lung cancer in males. While a previous meta-analysis found a modest increased risk for lung cancer associated with asthma, the magnitude of the risk ratios varied widely by study [41]. Most previous studies of asthma and lung cancer did not account for negative confounding by chronic bronchitis and emphysema. Of two that did, one found an OR of 1.5 (95% CI = 1.0–2.2) for asthma and risk of lung cancer after adjusting for chronic bronchitis and emphysema [37]. This study was conducted in nonsmoking women, however, and is therefore not comparable to ours since we found an inverse association only among males. The other study was conducted in males and females and found an OR of 1.1 (95% CI = 1.0–1.2) for asthma only and 0.73 (95% CI = 0.65–0.83) for both asthma and hay fever and lung cancer mortality. Our results were unaffected by adjustment for additional smoking variables, including environmental tobacco smoke, and support several studies that found inverse associations with asthma, eczema, and hay fever [15], [40], [42]–[44]. Although previous studies of the time from diagnosis of asthma to diagnosis of lung cancer have been inconsistent [37]–[40], we found that asthma was consistently inversely associated whether it was diagnosed within 5 years or more than 15 years prior to lung cancer.

Several potential explanations have been hypothesized for an inverse association between asthma and lung cancer [45]. Asthmatics might avoid smoking and other deleterious exposures that could trigger their asthma symptoms. Avoidance of such exposures may subsequently decrease their risk of lung cancer. However, we carefully adjusted for smoking and saw no consistent trends for asthma by smoking status. Often asthmatics are administered allergy medications (antihistamines, decongestants, corticosteroids, bronchodilators, antibiotics, etc.) over long periods of time due to the chronic nature of asthma. Although the potential impact of these medications on lung carcinogenesis is unclear, antibiotics, for example, might eliminate lung pathogens postulated to increase risk of lung cancer, such as Chlamydia pneumonia [46]. Finally, the “immunesurveillance hypothesis” suggests that asthma may stimulate the immune system such that it is better able to detect and destroy cancer cells [45]. That the inverse association is limited to males may not be surprising since previous studies have found sex-specific differences in the prevalence and severity of asthma, possibly due to bronchial hyperresponsiveness, differential effects of tobacco, or hormone related differences [47]. While we are unsure why we see inverse associations in males, verification and follow-up in other settings is desirable.

Our study has clear strengths. It is population-based and achieved a very high participation rate among both cases and controls. The study questionnaires were administered by interviewers who underwent centralized training, ensuring that important demographic and risk factor information (e.g., age, smoking) was obtained as accurately and completely as possible. In addition, quality control procedures were built into all facets of data collection and transfer [17].

These results must also be interpreted in the light of the study's limitations. Despite the large initial sample size, small numbers limited some sub-analyses (e.g., in never smokers). Given that previous lung disease exposure was ascertained through self-report, recall bias is possible. However, cases did not report all previous lung diseases at a consistently higher level than controls, as shown by the positive associations of chronic bronchitis and emphysema with lung cancer compared to the negative association for asthma. In addition, self report-based COPD was strongly associated spirometry-based COPD in a subset of cases with spirometry data, supporting a previous validation study that found self-reported COPD sufficiently robust for accurate estimation of relative risks [48]. While the prevalence of COPD varies notably in the published literature, the prevalence of COPD in controls in EAGLE (6.9%) was similar to the pooled prevalence of COPD in Europe (7.4%) [20]. In addition, the prevalence of asthma in EAGLE controls (4.7%) was similar to that reported in a general population study of northern and central Italy (3.3–5.5%) [49]. This similarity suggests little potential for selection bias in EAGLE, consistent with the careful protocol for population control accrual in EAGLE. Although surveillance bias (i.e., increased lung cancer diagnosis in individuals with COPD due to increased medical investigations such as chest X-rays) is theoretically possible, the differences in the magnitude and direction of associations for chronic bronchitis and emphysema versus asthma and the stability or increased strength of the findings over time, as demonstrated through latency analysis, argue strongly against this interpretation. The consistency of our results with the previous COPD literature is a further indication of their validity.

In one of the largest studies of COPD and lung cancer to date, we verified the associations of chronic bronchitis, emphysema, and COPD with lung cancer. Our extensive analyses of these associations by smoking status suggest that a component of the associations is independent of smoking. This independent association of COPD with lung cancer risk could potentially arise from chronic inflammation. The inverse association with asthma is consistent with some previous observations, and its restriction to men might explain some of the inconsistency in the literature. Further investigations in studies powered to evaluate men and women separately are warranted.
